# Realization of the right to adequate food and the nutritional status of land evictees: a case for mothers/caregivers and their children in rural Central Uganda

**DOI:** 10.1186/s12914-018-0162-6

**Published:** 2018-05-24

**Authors:** Aziiza Nahalomo, Per Ole Iversen, Peter Milton Rukundo, Archileo Kaaya, Joyce Kikafunda, Wenche Barth Eide, Maritha Marais, Edward Wamala, Margaret Kabahenda

**Affiliations:** 1 Department of Food Technology and Nutrition, College of Agricultural and Environmental Science, Makerere University, Kampala, Uganda; 2 Department of Nutrition, Faculty of Medicine, University of Oslo, P.O. Box 1046 Blindern, 0317 Oslo, Norway; 3 Department of Haematology, Oslo University Hospital, Oslo, Norway; 4 Division of Human Nutrition, Faculty of Medicine and Health Sciences, Stellenbosch University, Cape Town, South Africa; 5 Department of Human Nutrition and Home Economics, Kyambogo University, Kampala, Uganda; 6 Department of Philosophy, College of Humanities and Social Sciences, Makerere University, Kampala, Uganda

**Keywords:** Children, Food insecurity, Land evictions, Mothers/caregivers, Nutritional status, The human right to adequate food, Uganda

## Abstract

**Background:**

In developing countries like Uganda, the human right to adequate food (RtAF) is inextricably linked to access to land for households to feed themselves directly through production or means for its procurement. Whether RtAF is enjoyed among Ugandan land evictees, is unknown. We therefore explored this among land evictees (rights-holders) in Wakiso and Mpigi districts in rural Central Uganda. We assessed food accessibility and related coping strategies, diet quality and nutritional status of children 6–59 months old, and their caregivers. Effectiveness of the complaint and redress mechanisms in addressing RtAF violations was also explored.

**Methods:**

In this cross-sectional study, quantitative data was collected using a structured questionnaire, with food security and nutritional assessment methods from a total of 215 land evictees including 187 children aged 6–59 months. Qualitative data was collected by reviewing selected national and international documents on the RtAF and key informant interviews with 15 purposively sampled duty-bearers. These included individuals or representatives of the Uganda Human Rights Commission, Resident District Commissioner, Sub-county Chiefs, and local Council leaders.

**Results:**

We found that 78% of land evictees had insufficient access to food while 69.4% had consumed a less diversified diet. A majority of evictees (85.2%) relied on borrowing food or help from others to cope with food shortages. Of the 187 children assessed, 9.6% were wasted, 18.2% were underweight and 34.2% were stunted. Small, but significant associations, were found between food accessibility, diet quality, food insecurity coping strategies; and the nutritional status of evictees. We observed that administrative, quasi-judicial and judicial mechanisms to provide adequate legal remedies regarding violations of the RtAF among evictees in Uganda are in place, but not effective in doing so.

**Conclusion:**

Land eviction without adequate legal remedies is a contributor to food insecurity and undernutrition in rural Central Uganda. It is essential that the Government strengthens and enforces the policy and legal environment to ensure adequate and timely compensation of evictees in order to reduce their vulnerability to food insecurity.

**Electronic supplementary material:**

The online version of this article (10.1186/s12914-018-0162-6) contains supplementary material, which is available to authorized users.

## Background

The human right to adequate food (RtAF) is established in the International Covenant on Economic, Social and Cultural Rights (ICESCR), Article 11 which says *“The States Parties to the present Covenant recognize the right of everyone to an adequate standard of living for himself and his family, including adequate food, clothing and housing, and to the continuous improvement of living conditions*” [1]. The UN Committee on Economic, Social and Cultural Rights (CESCR) interprets, through its General Comment No. 12 (GC12) on the Right to adequate food, this provision to mean that the RtAF is realized “*when every man, woman and child, alone or in the community with others, have physical and economic access at all times to adequate food or means for its procurement*” [2], thus all citizens are referred to as rights-holders under international human rights law to which Uganda is a party. Additionally, realization of the RtAF requires the recognition of the interdependency and progressive realization of all human rights [2]. The Voluntary Guidelines to support the progressive realization of the RtAF in the context of national food security (VGRtAF or “Right to Food Guidelines”) urges State agencies as primary duty bearers to provide legal remedies to individual whose right to adequate food has been violated [3].

Developing countries face challenges in implementing measures aimed at ensuring, progressively, that their citizens will enjoy the RtAF. Food insecurity is typically caused by problems such as poverty and vulnerability, lack of political and financial commitment, inadequate resources, climate change, unawareness of the RtAF by both duty-bearers and rights-holders, and external shocks to families such as HIV/AIDS [4]. Uganda faces similar challenges as other developing countries; however, the realization of the RtAF for some vulnerable communities in Uganda is further threatened by inadequate access to land, land tenure systems that do not favour the poor, and the increasing land evictions [5–7].

Access to land is essential for the rural populations in developing countries because agricultural production is often their major livelihood option. In low income, subsistence-dependent economies, peasants rely heavily on access to land to feed themselves and their families, through directly consuming the food produced and/or through income generating activities that allow the purchasing of food [8]. In developing countries like Uganda where over 70% of the population is engaged in agriculture as the main source of livelihood and income [9] and 43.3% rely on subsistence agriculture [10], access to land is essential for people to procure adequate food. Hence it is important to assess the effects of land evictions on the affected populations’ food and nutrition security. Land evictions may lead to individuals, households and communities becoming either fully or partially landless and consequently depriving them of their RtAF [11].

The hike in land sales is also blamed for the increasing landlessness, especially in rural areas of Uganda [12]. Given the context of land fragmentation and as Uganda tries to transform agriculture from a subsistence to a commercial form, it is becoming increasingly common to evict people from land which they depend on for their own food production and livelihood [12–14]. In Uganda, land evictions have occurred in different parts of the country, however they become rampant between 2006 and 2014 [15–17], leaving many people landless and homeless [18]. Evictions are justified in the name of promoting area development through creation of commercial farms, residential houses or estates and new infrastructures such as industries, schools and roads that will offer employment opportunities and other basic services to the local natives [19]. However, the intended developments may not benefit the actual land evictees especially where access to appropriate legal or other forms of protection are denied. In addition, States are urged by the Voluntary Guidelines on the Responsible Governance of Tenure of Land, Fisheries and Forests in the Context of National Food Security (VGGT) to ensure tenure security which guarantees legal protection against forced evictions that are inconsistent with States’ existing obligations under national and international law, and against harassment and other threats [20].

Although the law, under Section 237 of the Ugandan Constitution, acknowledges customary land ownership and guarantees protection of rights to all types of land ownership [21], some citizens are evicted from customary land without due process, consultation and adequate notice or compensation [22]; hence rendering the evictees vulnerable to food insecurity. In line with this, land evictions also negatively impact household food security because the evictions or negotiated transfers often involve the transfer of high-value land from the poorer population to middle or upper-income groups, and the freeing of land to set up different infrastructures does not often benefit the poor [23, 24]. Subsequently land evictions are becoming a public health concern because limited or lack of land-ownership is linked to about 80% of cases of hunger and undernutrition among people that live in rural areas [25]. Moreover, land evictions also limit the evictees’ ability to access clean water, food, proper sanitation, health and education [22].

Whereas the Government of Uganda (GoU) has enacted legislations protecting people from land evictions [21, 26], these evictions, without adequate compensation, continue in various parts of the country [18] and this is denying peasant households a chance to access and use land to ensure their food security. The case of evictees without compensation shows the close interrelations between the rights to land, to adequate housing, food and health.

In addition to the land eviction problem, Uganda is still ranked among the countries that are highly burdened by hunger and undernutrition [27, 28]. Nearly one-third of children are stunted due to chronic undernutrition, 11.5% are underweight while wasting (a life-threatening acute undernutrition condition) is at 4% [29, 30]. Moreover, the 2014 Census revealed that the majority of Ugandans consume a sub-optimal number of meals per day; 51.4% had consumed two meals, while 12% had consumed only one meal, with the problem being more pronounced in the rural areas [31]. Anemia, a debilitating health condition mostly linked to poor diet and micronutrient deficiencies, is a severe public health concern. In the last 5 years levels of anemia increased in children from 49 to 53%, and in women of reproductive age from 23 to 33% during the same period and this is attributed to poverty, poor dietary intake of iron and malaria infections [29].

Land evictions are known to interfere with the rights-holders’ enjoyment of the RtAF. With the increasing land sales in the study region, it is important to examine whether in the presence of evictions, evictees are enjoying their human right to adequate food. The aim of the study was therefore to investigate the realization of the human right to adequate food among cases of land evictees (rights-holders) in rural Central Uganda. Specifically we wanted to determine the level of food accessibility, diet quality, food insecurity coping strategies and the nutritional status of children (6–59 months) and their caregivers (18–49 years) and the effectiveness of the complaint and redress mechanisms in addressing right to adequate food violations among evictees.

## Methods

### Design and sampling procedure

This cross-sectional study was performed in Wakiso and Mpigi districts in rural Central Uganda in 2013. Rights-holders were adult mothers (18–49 years) or other primary caregivers of children (6–59 months) from households that had been evicted from land in these districts. We included these two groups of evictees because they are often more vulnerable to effects of food insecurity than e.g. adult men [32]. We also selected primary duty-bearers who could serve as key informants.

### Identification and inclusion of evicted households and selection of duty-bearers

The process of identifying the rights-holders (land evictees) involved:

- Reviewing of literature and new reports to get information about areas where land evictions had occurred.
- Mapping out areas reported to have faced land evictions by site visits with the help of local area leaders such as local council leaders, community development officers, and religious leaders.
- Validation of evicted areas through inquiries from the local area leaders and the local residents of the mapped out areas.
- Identification and enlisting of individual households of rights-holders in consultation with local area leaders. Owing to absence of an up-to-date list of all evicted households in the respective mapped out research areas, this step also involved allocation of a unique identification number to each listed evictee household.

We included households that had been evicted from land within the district and settled within the same district of study. These households had to be located by the researcher during the mapping exercise. The household members were asked for consent to participate in the study. In addition, 15 duty-bearers (key informants) were purposively selected among representatives of institutions considered as having the mandate of promoting the realization of the RtAF among evictees in the study areas.

### Identification and ranking of food insecurity coping strategies used by evictees

Focus group discussions (FGDs) in the study area with the purposively selected four focus groups of six to sixteen participants following the standard methodology described by Maxwell and Caldwell [33] were held to identify and rank the food insecurity coping strategies used among land evictees. This involved:

- Identification and listing of food insecurity coping strategies used in the study area and this was composed of 18 strategies.
- Consultation about the focus groups’ perceptions of the severity of each of the listed strategy ranging from very severe, severe, moderate, and least severe.
- Ranking the severity of each strategy by each of the focus groups from a scale of 1 to 4, where 1 indicates the least severe strategy; 2 indicates moderate, 3 indicates severe while 4 indicates the most severe strategy.
- Calculating the average severity ranking of each strategy from each of the four focus groups, which were then combined with the frequency of each coping strategy to calculate the coping strategy index score for each household that was included in the study.

### Sample size determination

Land evictees were enlisted from the purposively selected seven villages where a total of 574 households were identified during the mapping exercise. The Emergency Nutrition Assessment for Standardized Monitoring Assessment Relief Transitions software was used to calculate the sample size as:

$$ \mathrm{n}=\frac{1.96^2\mathrm{x}\kern0.5em \mathrm{p}\kern0.5em \mathrm{x}\left(1-\mathrm{p}\right)\left[\mathrm{x}\kern0.5em \mathrm{DEFF}\right]}{{\mathrm{d}}^2} $$

Where:

n = sample size

p = malnutrition prevalence

d = minimum acceptable precision level (confidence level)

DEFF = Default design effect.

By computation, the total number of evicted households located during the mapping exercise was 574. The percentage of child stunting (a proxy for malnutrition) in the study area was 27.7 [30]. Level of statistical significance (*p*-value) was 5% and the default design effect was 1 because the population was homogenous and found within a small area (sub-county) as recommended by Part B of the Guidelines on Nutritional Survey Methodology in Uganda [34]. In order to cater for the unforeseen contingency, the sample size (*n* = 201) was increased by 10% to 221 households that were randomly selected to this study. The number of households selected from each village was proportionate to the total number of evictee households in that area. Since the evicted households (HHs) were considered as the measurement unit, one household member (a mother or primary female child caregiver) acted as a respondent for the selected household.

### Assessment of food accessibility

Food accessibility among land evictees was determined using the Household Food Insecurity Access Scale (HFIAS) following the standard methodology described by Coates et al. [35]. The HFIAS has nine generic questions across the three domains of anxiety and uncertainty about household food supplies, insufficient food quality and insufficient food intake. Each question within the questionnaire had a ‘Yes’ and ‘No’ option. If the respondent answered ‘No’ to the question, the household score was ‘0’. The ‘Yes’ option had three alternatives; (i) rarely, scoring 1; (ii) sometimes, scoring 2; and (iii) often, with a score of 3. If the household response to all the nine questions was ‘often’, the maximum score for a household was 27 and a minimum of 0 if the respondent answered ‘No’ to all the questions. The generated score from 0 to 27 reflected a single statistical dimension of food accessibility. The higher the score, the more households were unable to access food and the lower the score, the less unable the household was to access food. HFIAS scores were correlated with anthropometric measures (BMI, z-scores for weight-for-age, height-for-age, and weight-for-height) to determine associations between food accessibility and evictee nutrition outcomes.

### Assessment of household diet quality

The nutritient adequacy of the diets of land evictees was determined using the Individual Dietary Diversity Score (IDDS) tool as described by FAO [36] based on 14 food groups and a reference period of 1 day. Using the IDDS questionnaire, adult females or caregivers in charge of food preparation were asked to report their consumption of foods or drinks in the previous day during the day and at night including foods or drinks consumed outside home. Questions probing the description of the food or drinks consumed as breakfast, midmorning snack, lunch, evening snack and supper or dinner were asked and the respective responses were recorded. In case a particular meal was not consumed, the respective section would be skipped. After completion of the respondent’s recall, recording in the questionnaire of all foods or drinks eaten under each respective food groups was done and for any missing food group, the respondent was asked if any food item in that group was eaten. The IDDS was then calculated by summing the different food groups consumed by each respondent over 24-h period. Food quantities of approximately one tablespoon or less (< 15 g) were not included in the score as recommended by Arimond and colleagues [37]. Minimum score was 0 if the household did not consume any food group and the maximum score was 14 if the household consumed all the food groups. The higher the score indicated a higher nutrient adequacy of the diets consumed by land evictees while the lower the score indicated a lower nutrient adequacy of their diets. The IDDS were correlated with anthropometric measures (BMI, z-scores for weight-for-age, height-for-age, and weight-for-height) to determine associations between dietary adequacy and evictee nutrition outcomes.

### Assessment of food insecurity coping strategies

Food insecurity coping strategies among land evictees were determined using the Coping Strategy Index (CSI) following the standard methodology described by Maxwell and Caldwell [33]. The adult females (mothers or caregivers) with the primary responsibility for preparing and serving meals were asked a series of questions regarding how their respective households responded to food shortage in the last 7 days preceding the survey. The relative frequency of how many days per week a household had to rely on the various coping strategies ranking from “never” to “every day” was then recorded. The CSI scores for each household were calculated by combining both the frequency and the severity of each of the coping strategies [33], as ranked by the four focus group discussions conducted before actual data collection. Minimum score was 0 if the household did not use any food insecurity coping strategy and maximum was 126 if a household used all the food insecurity coping strategies everyday during the 7 days preceding the survey. The higher the CSI score is the higher the level of food insecurity in the household. The CSI scores were correlated with anthropometric measures (BMI, z-scores for weight-for-age, height-for-age, and weight-for-height) to determine associations between food insecurity and evictee nutrition outcomes.

### Assessment of land evictees’ nutritional status

Anthropometry was assessed following standard protocols by WHO [38] to estimate the nutritional status of land evictees. Weight and height of female adults were measured to determine risk of underweight based on BMI. If a household contained more than one eligible woman, the index caregiver was randomly selected using ballot papers. In households where there was no female caregiver, the available adult male child-caregiver was interviewed, but his anthropometric measurements were not taken. The cut-off points for classification of caregivers’ nutritional status were: underweight (BMI < 18.5 kg/m^2^), normal BMI (18.5–24.9 kg/m^2^), overweight (BMI ≥ 25.0 kg/m^2^) and obese (BMI ≥ 30.0 kg/m^2^) [39].

Weight was recorded to the nearest 0.1 kg using a UNICEF electronic scale. Light or minimal clothing on the child or caregiver was ensured. Children who were able to stand were weighed standing on the scale. Those who could not stand were weighed with their mothers or caregivers who were weighed holding the child and the weight of both was taken; the mother or caregiver was then weighed alone and the difference between the weights was equivalent to the weight of the child. Presence or absence of oedema for each child was also recorded since its presence may lead to elevated weight measurements [38].

Height or length was recorded to the nearest 0.1 cm using a stadiometer or length board. Recumbent length for children below 2 years and children who were unable to stand was taken. Height for children above 2 years and adult females was taken while standing on the stadiometer. The anthropometric data was then processed using WHO Anthro version 3.2.2 [40] to generate length-for-age (LAZ), height-for-age (HAZ), weight-for-age (WAZ) and weight-for- height (WHZ) z scores. For each indicator, the cut-off used to determine nutritional status was based on WHO guidelines (2006), where values < − 3 was categorized as severely undernourished, − 3 to − 2 as moderately malnourished, and ≥ − 2 as having a normal nutritional status.

### Assessment of effectiveness of the complaint and redress mechanisms regarding violations of the RtAF among land evictees

Effectiveness of the complaint and redress mechanisms in addressing violations of the RtAF among land evictees was assessed through document analysis and analysis of qualitative data obtained from the key informants (duty-bearers) and the land evictees (rights-holders). Key interview guides were used to obtain data from the key informants (Additional files 1 and 2). Structured questionnaires with open ended questions were used to obtain data from the land evictees regarding the availability, awareness, accessibility, usage and adequacy of the complaint and redress mechanisms provided in case of RtAF violations resulting from land evictions (Additional file 3).

### Data analysis

Statistical analyses were conducted using SPSS (Statistical Package for Social Scientists) version 17. For most variables, proportions of individuals or households were computed to find the frequency of malnutrition and food insecurity among the land evictees. Spearman’s correlation coefficient (r_s_) was computed to determine if associations were observed between food accessibility, diet quality, food insecurity coping strategies and the nutritional status of evictees. *P*-values < 0.05 were considered statistically significant. Qualitative data was transcribed and included in the results and discussion of results.

## Results

### Socio-demographic characteristics of land evictees

Out of the expected 221 evictee households, 218 agreed to participate. Three questionnaires were incomplete and thus not included. Full response was obtained from 215 evictees (rights-holders). More than 55% of the study households were male headed while 43% were headed by females. Most households had a large number of members (the median was 7) and most of the evictees (81.5%) were born within the sub-counties where they had resettled. It was important to note that while the majority of households (70.2%) suffered from partial land evictions, only about 1/3 of the evictees were displaced from all the land that they initially possessed (Table 1). The median duration of displacement after evictions was 8 months.

**Table 1:** Demographic and socio-economic characteristics of the 215 households

Characteristic	Number	Percentage
Head of the household		
Male	123	57.20
Female	92	42.80
Birth place		
Born in the area	175	81.39
Not born in the area	40	18.61
Household size		
2–4	30	13.95
5–7	79	36.74
8–10	94	43.72
11–14	12	5.59
Type of eviction		
Part of the land	151	70.23
All the land	64	29.77
Duration of eviction (months)		
2–4	50	23.25
5–7	40	18.60
8–10	68	31.62
11–13	34	15.81
14–17	23	10.72

### Land evictees’ food accessibility

Despite the fact that 70.2% of the evictees had only been partially evicted from their land, there were high levels of insufficient access to food among land evictees. More than 3/4 of the households were worried about their families not having enough food to eat (Table 2). About 2/3 reported limited food variety and consumption of less preferred foods such as maize porridge, maizemeal (locally referred to as posho), cassava, sweet potatoes and tea without milk. These land evictees also reported that they regularly consumed maize flour that were past the expiry date and were often moldy, insect infested and with a bad smell. Evictees also indicated that they often bought poor quality flour despite its bad smell and bad taste. Additionally, 6.3% reported spending the whole day and night without eating any food for at least once in the last 30 days preceding the survey.

**Table 2:** Proportion of the 215 households that had insufficient access to food during the previous 30 days

Indicators of insufficient food accessibility	Percent
I. Anxiety and uncertainty about household food supplies	
1. Worried that their households would not have enough food to eat	78.40
II. Insufficient food quality (food variety and preferences)	
1. Were not able to eat the kinds of foods they preferred	52.10
2. Had to eat a limited variety of foods	68.90
3. Had to eat some foods that were not wanted	62.60
III. Insufficient food intake (quantity of food)	
1. Had to eat smaller meals than needed	47.30
2. Had to eat fewer meals in a day	53.10
3. Had ever had no food of any kind to eat in the household	30.50
4. Had to go to sleep at night hungry	11.00
5. Had to go a whole day and night without eating anything	6.30

Based on the three domains of food inaccessibility (Table 2), 78.4% reported feelings of uncertainty or anxiety about household food supplies, 61.2% relied on food of insufficient quality while 29.6% relied on food of insufficient quantity.

### Adequacy of the land evictees’ diets

**Fig. 1:**
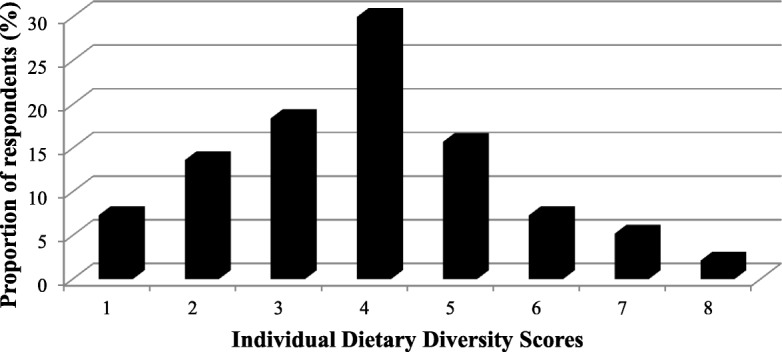
Dietary diversity scores of land evictees’ diets (*n* = 215)

Results of the dietary adequacy of the evictees as assessed using the IDDS are presented in Fig. 1. The mean (±SD) IDDS was 3.9 ± 1.6 out of the 14 food groups. The majority of evictees (69.4%) reported a low dietary diversity score (range 1–4), 23% reported a medium dietary diversity score (5–6), and only 7.3% reported a high dietary diversity score (7–8).

**Fig. 2:**
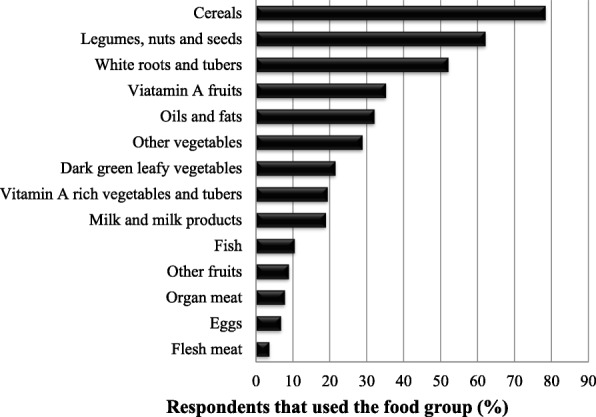
Proportion of land evictees (*n* = 215) that consumed the individual food groups in last 24 h before the survey

In general, the land evictees’ diets were comprised of nutrient-dilute staple foods and relishes. Figure 2 reveals that cereal-based foods, legumes, nuts and seeds and the white roots and tubers, were the food groups from which land evictees selected foods consumed the day preceding the survey. Consumption of animal-source foods was also very low.

**Fig. 3:**
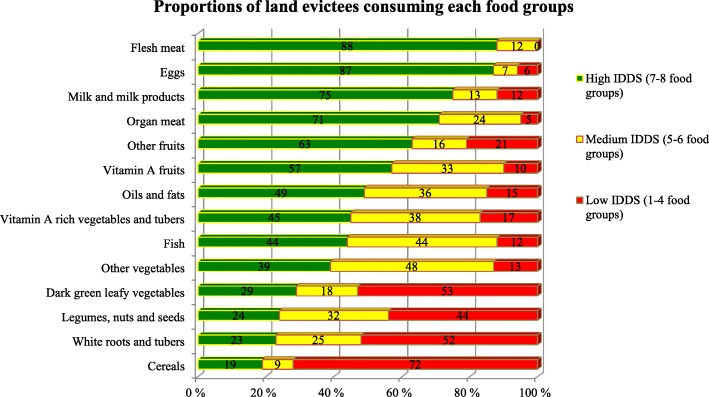
Proportions of land evictees (*n* = 215) consuming various food groups. Data are presented as dietary diversity tertiles (i.e. 1–4; 5–6; and 7–8 food groups)

Out of the possible score of 14, the evictees scored between 1 and 8, the corresponding tertiles are given in Fig. 3. Analyses according to these dietary diversity tertiles, revealed that inclusion of fruits, vegetables, animal-source foods, and fat and oil improved dietary diversity. Land evictees with the lowest dietary diversity were those who had consumed cereals (maizemeal, maize porridge and rice), legumes and nuts such as beans and milled groundnuts and white roots and tubers such as white sweet potatoes and cassava. Conversely, households with high dietary diversity had included fats and oils, vegetables, fruits and some animal-source foods (milk, eggs, meat and fish) in their diets.

### Food insecurity coping strategies used by land evictees

**Fig. 4:**
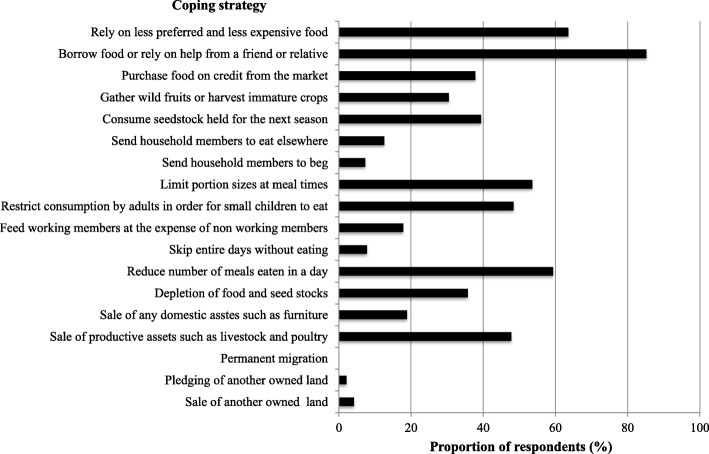
Coping strategies commonly used by land evictees (*n* = 215) to cope with food insecurity

Generally, several coping strategies were used by land evictees to mitigate food insecurity. Figure 4 revealed that the majority (85.2%) of land evictees relied on borrowing food or relying on help from others to cope with food insecurity. The other commonly used coping strategies were relying on less preferred and less expensive foods (*n* = 137), reducing the number of meals (*n* = 128), and food portions (*n* = 115). More than 18% sold off their domestic and productive assets while 4.2% sold off their other owned land to mitigate food insecurity.

### Nutritional status of land evictees

A total of 187 children (0–59 years) from 215 evicted households were assessed to ascertain their nutritional status (Table 3). Overall, the children exhibited some undernutrition. The results indicate that one in ten children were wasted, almost one fifth were underweight and slightly more than one third were stunted (Table 3).

**Table 3:** Nutritional status of children (6–59 months) from evicted households

Percentage of children (6–59 months) classified as undernourished based on anthropometric z-scores (*n* = 187)^a^									
	Weight-for-height			Weight-for-age			Height-for-age		
	% < -3SD	% < -2SD	Z-score^b^	% < -3SD	% < -2SD	Z-score	% < -3SD	% < -2SD	Z-score
Age group (months)									
6–11 (*n* = 27)	0.0	7.4	−0.1 ± 1.0	6.1	25.9	−0.6 ± 1.2	11.1	44.4	0.9 ± 1.5
12–23 (*n* = 72)	1.4	12.5	−0.4 ± 1.1	4.2	16.7	−1.0 ± 0.9	4.2	38.8	−1.1 ± 1.2
24–35 (*n* = 43)	2.3	13.9	−0.4 ± 1.1	6.9	9.3	−0.9 ± 1.1	6.9	30.2	−1.1 ± 1.1
36–47 (*n* = 28)	3.6	3.6	−0.2 ± 0.9	0.0	14.3	−0.7 ± 0.8	3.6	21.4	−0.9 ± 1.0
48–59 (*n* = 17)	0.0	0.0	−0.1 ± 0.8	5.8	11.7	−0.9 ± 1.0	11.7	29.4	−1.3 ± 1.0
Total % of children	1.6	9.6	−0.3 ± 1.0	3.2	18.2	0.8 ± 1.0	6.4	34.2	−1.1 ± 1.2

^a^Total children with valid dates of birth (month and year of birth) and with complete weight and height measurements

^b^Values are mean ± SD

There was a significant proportion of both underweight and overweight among the caregivers. The mean (±SD) BMI was 22.2 ± 3.7 kg/m^2^. About a quarter of the caregivers (26.9%) were underweight, 11.7% were overweight while 5.9% were obese.

### Associations between nutritional status and food insecurity

In general, there was a small significant association between nutritional status and food insecurity as depicted by the r_s_ values. The association of undernutrition among both caregivers and their children were linked to their food security status. The association of BMI among women were related to limited food access (r_s_ = 0.29; *p* = 0.004) and limited dietary diversity (r_s =_ − 0.02; *p* = 0.003; Table 4). Conversely, the association of stunting and underweight among children were negatively related to food accessibility (r_s_ = − 0.32; *p* = 0.002 and r_s_ = − 0.19; *p* = 0.003, respectively; Table 4), which suggests that children were not getting adequate food to maintain their growth. Stunting was also associated to the number of strategies employed by the household to cope with food insecurity (r_s =_ − 0.11; *p* = 0.004); which indicates that households with severe food insecurity engaged in multiple strategies to cope with the food insecurity, but were still not enjoying their RtAF, hence the chronicity of hunger among evictees is evidenced by chronic undernutrition (stunting) among children.

**Table 4:** Associations between nutritional status of land evictees and household food security indices

Nutritional status	Association with food security status					
	HFIAS Score	*P*-value	IDDS	*P*-value	CSI Score	*P*-value
Women (18–49 years) (*n* = 204)						
BMI	0.29	0.004	−0.02	0.003	0.06	0.140
Children (6–59 months) (*n* = 187)						
Wasting	−0.07	0.279	0.02	0.020	−0.08	0.286
Underweight	−0.19	0.003	0.03	0.002	−0.04	0.140
Stunting	−0.32	0.002	0.02	0.009	−0.11	0.004

Values are Spearman’s correlation coefficients

Wasting (low WHZ), which is an indicator of acute undernutrition, was not associated with food accessibility (HFIAS). Overall, better diet quality as measured by dietary diversity (high IDDS) was positively associated with reduced risk of child undernutrition indicated by stunting (low HAZ), underweight (low WAZ) and wasting (low WHZ).

### Effectiveness of the complaint and redress mechanisms regarding violations of the RtAF

Generally, it was established that Uganda has several administrative, quasi-judicial and judicial mechanisms to provide adequate remedies regarding violations of the RtAF. However, the mechanisms were not accessed by the majority of land evictees; furthermore the remedies provided to the few evictees who accessed the complaint and redress mechanisms, did not correspond to the damages incurred to evictees after evictions.

We established that in Uganda, justiciability of the RtAF is seconded by Article 45 of the 1995 Constitution of Uganda stating that *“*The rights, duties, declarations and guarantees relating to the fundamental and other human rights and freedoms specifically mentioned in this Chapter shall not be regarded as excluding others not specifically mentioned*”* [21]. This means that Uganda is bound by international obligations on the RtAF whether or not these are incorporated in domestic law. At the national level, Uganda has the Land Division of the High Court that deals with all land issues, and the Uganda Human Rights Commission (UHRC) which is mandated by the 1995 Uganda Constitution to protect and promote all human rights in Uganda. At the local level, the available quasi-judicial mechanisms were found to be the District Land Board, the Area Local Council leaders (LC I, II, III and V), the parish court, the sub county court, the Chief Magistrate’s Court, the area police officers and the Resident District Commissioners. However, in some instances, rights-holders may directly address their complaints to their area member of parliament or area minister. It is then the responsibility of these local quasi-judicial mechanisms to proceed with taking the land evictees’ complaints to court or relevant authority as reported by some of the key informants. When the conflict is not solved by the LC1, then the case is referred to the parish court, the sub-county court and finally to the Chief Magistrate’s Court which has supervisory roles over the LC courts.

Among the total evictees, 161 (74.8%) were aware of some of the existent complaint and redress mechanisms in case of any violations related to food arising from land evictions (Table 5). However, only 62 (38.4%) of evictees reported to have accessed and used the available recourse mechanisms. Among the evictees who had accessed and used the existent complaint and redress mechanisms, the majority of evictees 37 (59.6%) reported their complaints to the Area Local Council leaders.

**Table 5:** Land evictees’ awareness, accessibility and usage of the available recourse mechanisms

Variable	Number	Percentage
Awareness of existent redress or complaint mechanisms available for land evictees (*n* = 215):		
Yes	161	74.88
No	54	25.12
Usage of any of the complaint or redress mechanisms after land evictions (*n* = 161):		
Yes	62	38.50
No	99	61.50
Authority/organ/individual reported to (*n* = 62):		
Local council leaders	37	59.67
Parish or Sub county courts	12	19.35
Resident District Commissioner	0	0.00
District Land Officers	0	0.00
Area police officers	10	16.12
Others (Area Member of Parliament)	3	4.86

### Land evictees who received remedy, assistance or reparation after land evictions

Table 6 reveals that 54 (87.6%) of evictees reported not to have received reparation after land evictions while only 3.2% reported to have received adequate assistance or reparation received. However, 9.6% did not know whether the assistance or reparation received was adequate or not. Cash transfers of between 100.000–500.000 Uganda shillings (equivalent to about 38–194 USD) were reported as the most commonly received type of assistance or reparation by 70.2% of land evictees. Additionally, as reported by one of the key informants, there was no appropriate legal procedure upon which the received cash transfers were decided upon. Furthermore, the cash transfers received were not equivalent to the value of the land and property lost, as noted by another informant:

*“Some of the evictees were given a small compensation from the evictors in form of money of less than 600,000 Uganda shillings, which was decided by the evictors without consulting the evictees. That money cannot be equated to the value of the property and assets lost by the evictees”.*

**Table 6:** Land evictees who received assistance or reparation after land evictions

Variable	Number	Percentage
Received adequate assistance or reparation (*n* = 62):		
Yes	2	3.22
No	54	87.09
Do not know	6	9.71
Type of assistance or reparation received (*n* = 37):		
Food transfers	0	0.00
Cash transfers	26	70.27
Given alternative land	10	27.02
House construction	1	2.71
Non repeatition	0	0.00
Apology	0	0.00
Evictor taken to court or prison	0	0.00

### Percentage of evictees adequately resettled or rehabilitated

At the time of conducting this research, no land evictee reported to have been adequately resettled or rehabilitated. In addition, as reported by one of the key informants: *“since there is no resettlement policy in Uganda, the displaced individuals always cater for their new settlements immediately after land evictions. Some opt to live with their relatives or friends while others migrate to urban areas to search for a new source of living”.*

### Right to adequate food complaints filed, investigated and adjudicated in court or other relevant institutions

The respondent at UHRC reported that there were no records of land evictees who had so far sought legal redress for violations of their RtAF partly because they are unaware of the provisions on the justiciability of this right and also not acquainted with offices or institutions where such cases are handled.

### RtAF claimants affected by land evictions benefiting from legal aid

The respondent at UHRC reported that no legal assistance was extended to land evictees to seek legal redress for violation of their RtAF. This was in agreement with the information from one of the key informants which further clarified that: *“no legal personnel have ever contacted the land evictees to offer them any legal help of any kind ever since the land evictions occurred”*.

### Programmes to support land evictees in case of failure to acquire adequate food

As reported by some key informants there were no food transfer schemes targeting land evictees. Food packages could be organized temporally and distributed among land evictees (as was the case with land evictees in Kayunga District-Central Uganda) only after the Minister of Lands, Housing and Urban Development had reported on the situation to the President of Uganda who could then order for the food to be distributed among land evictees. However, this was not the case with land evictees in the study area.

Additionally, there were no resettlement programmes targeting land evictees in the study area. Resettlement is left in the hands of evictees themselves, as reported by one key informant.

## Discussion

Where people depend on land for their food security, secure land rights are essential to the progressive realization of the RtAF [41]. This study revealed high levels of insufficient access to food among land evictees as indicated by more than three quarters of households that had been worried about their families not having enough food to eat in the 30 days preceding the survey. This shows that the larger part of evictees who were unable to access sustainable food supplies due to lack of resources (e.g. lack of enough money or inability to grow or trade for the food) [35]. This study corroborates findings from farm dweller evictees in West Rand region in the province of Gauteng in South Africa who reported inability to access land after eviction which limited their own food production [42]. This may suggest that evictees’ financial costs associated with acquisition of food for an adequate diet were at such a level that the attainment and satisfaction of other basic needs were threatened by lack of resources. Additionally, inability to grow food due to evictions implies limited physical accessibility to food by evictees hence further increasing their vulnerability to food insecurity.

What is most worrying is the fact that about two-thirds of land evictees were coping with food insecurity by consuming less preferred foods such as moldy and insect-infested beans due to their inability to purchase better quality beans. Prolonged consumption of moldy and insect-damaged foods pose a risk of chronic disease such as cancer and infections [43], which may further compromise the health and nutritional status of land evictees. This practice is also not in tune with paragraphs 10 and 11 of GC12 which emphasizes the importance of assuring food safety and the perceived non nutrient-based values attached to food and food consumption as essential for the realization of the RtAF [2].

This study revealed that some land evictees resorted to extreme food insecurity coping strategies such as ‘*plegding and sale of another owned land’ and* ‘*sale of domestic and productive assets’* in the 4 weeks preceding the survey. Sale of assets suggests more long term consequences that would further impact on the quality of life and possibly food security of the evictees. Surprisingly, the levels of impaired linear growth (stunting) and thinness (underweight and wasting) are not so different from the regional and national levels reported in the Uganda Demographics and Health Survey [30]. This may suggest that in a region already burdened by poverty, hunger and undernutrition [27, 28], presence of evictions without the provision of, and access to, appropriate forms of legal or other protection will further interfere with the ability of evictees to enjoy the RtAF.

The RtAF involves ensuring “access to the minimum essential food which is sufficient, nutritionally adequate and safe, …” [2] and dietary diversity is often used as a proxy measure for nutrient or dietary adequacy for individuals [36]. The majority of land evictees engaged in this study reported to have used four food groups or less, which indicates that most diets were nutritionally inadequate [44]. In addition to low quality, the high proportion of households that reported ‘*borrowing food or relying on others*’, ‘*reducing the number of meals*’, ‘*reducing amount of food apportioned to household members*’ and ‘*restricting adult consumption’* suggests that the food was also limited in quantity. Prolonged intake of less diverse diets, especially when coupled with reduced amounts of food and/or eating occasions, emerged as a major concern because this may lead to both macronutrient and micronutrient deficiencies and as well as increased slower growth and malnutrition amongst children < 5 years among land evictees. In general, there was lower intake of animal-source foods among land evictees with the lowest dietary diversity (IDDS ≤4); which may be attributed to lack of or reduced finances and/or diversion of finances to meet other basic needs in the process of resettling or readjusting their living situations. GC12 obliges States that have ratified the ICESCR to respect, protect and fulfill the citizens’ RtAF [2]. The obligation to respect the RtAF, means that the State should not arbitrarily interfere with people’s RtAF or make it difficult for them to gain access to food, while the obligation to protect means that the State must pass and enforce laws to prevent third parties from violating this right. The obligation to fulfill (facilitate and/or provide) means that the State is required to take positive actions to identify vulnerable groups and to implement policies to ensure their access to adequate food is facilitated, however when people are faced with circumstances beyond their control, then the State should provide food directly to them [45].

Results of this study further show that the majority of land evictions involve losing rights to part of their land leaving them on or with new small plots of land as compensation. These small plots or portions of land are often insufficient to carry out farming as they have traditionally practiced it; hence there is need to support the evicted families for them to enjoy their RtAF. In addition, the migrations resulting from land evictions are also associated with increased migration costs and reduced agricultural productivity [46], which may negatively affect household food security. The State has the obligation to protect which requires measures by the State to ensure that enterprises or individuals do not deprive individuals of their access to adequate food [47]. The State needs to improve the process of evictions and provide remedies to enable evictees achieve the ‘right to feed themselves’. Inefficient use and performance of remedy mechanisms indicate that many land evictees in rural Central Uganda may not be enjoying their RtAF.

Food insecurity (limited food access) and poor diet quality (limited dietary diversity) were both associated with undernutrition among children; and this is likely to have long-term consequences on physical growth and cognitive development of these children. The prevalence of undernutrition among mothers’ or other caregivers was high according to the WHO (2010) undernutrition cut-off values for public health significance. This calls for interventions to reduce undernutrition among women in order to avert the consequences of poor maternal undernutrition for their children and future generations.

Like for most food insecure populations, several strategies were being employed to address food insecurity; however, changes in the diet that have resulted in the shift from using the common traditional banana plantain to the use of non-traditional, high-carbohydrate foods such as maize meal (posho) and maize porridge, pose a risk for overweight as observed among women.

Our results further show that Uganda has a conducive legal environment and an institutional framework through which evictees may as rights-holders seek complaint and receive redress mechanisms. Surprisingly about three-quarters of evictees are aware of the existence of complaint and redress mechanisms; however more than half of evictees neither complained nor sought legal remedies from these mechanisms. As reported by one key informant this may be attributed to lack of awareness of the constitutional provisions on the justiciability of the RtAF or not being acquainted with offices or institutions where such cases are handled. Guideline 7 of VGRtAF emphasizes the need to ensure access to effective remedies; including access to justice for victims whose RtAF has been violated [3]. In addition, paragraph 13 of GC12 calls for the need to have access to effective judicial or other appropriate remedies at both national and international levels in case of violations of the RtAF [2]. Victims of such violations are entitled to adequate reparation, which may take the form of restitution, compensation, satisfaction or guarantees of non-repetition [2]. However in this study, cash transfers were reported as the most received type of assistance or reparation by nearly one third of land evictees. Additionally, there was no appropriate legal procedure upon which the received cash transfers were decided upon as reported by one of the key informant. This questions the inability to apply human rights principles that should allow fair and just compensation for all losses incurred by the evictees [11].

Knowledge and awareness about the RtAF by primary duty-bearers is an essential pre-condition for the realization of the RtAF. Without knowledge, first of all among relevant state agencies, of the RtAF and the possibilities of remedies in the case of violations, a rights- holder may be deprived of this human right without knowing it [48]. Legal aid in relation to the RtAF comprises the financial or practical assistance extended to individuals who cannot afford to take legal action in order to realize their RtAF [48]. VGRtAF no. 1.5 further encourages States to assist individuals or groups of individuals to have access to legal aid to facilitate the progressive realization of the RtAF [3].

As Hospes [47] argues “*networking between and concerted action of policymakers, legal experts and civil society lobbyists”* are needed* “to facilitate implementation of the right to food and to address different obligations of states*” In the context of Mpigi and Wakiso districts, where the increasing land sales are coupled with evictions, there is need to sensitize both the public and land owners (and bonafide) on the correct processes of land transfers to ensure that the RtAF is protected. This calls for collaborative engagements among cultural institutions, non-governmental organizations, and local governments with oversight from the central government.

This study has some limitations, e.g. the inability to locate all evictees in the study area, as some of them immediately after evictions relocate to other areas to stay with relatives or friends or to search for other livelihood conditions. Additionally, the study period was short and not fully appropriate for determining the longer-term effect of eviction on the nutritional status (stunting and BMI) of the evictees. Moreover, any seasonal variations in food availability could not be determined and our data set did not permit statistical modelling of possible relations between indicators of nutritional status and food insecurity. Finally, we did not have a control community (control group) to provide baseline data to be compared with the results from the land evictees.

## Conclusions

This study shows that in Uganda; a country already burdened by malnutrition, poverty and vulnerability, the presence of land evictions without the provision of, and access to, appropriate forms of legal or other protection further hinders the realization of the RtAF among evictees. Despite the existence of the land fund under the Ministry of Lands and Urban Development, there is need for adoption of a policy to cater for land evictions and resettlements arising from irregular land sales, infrastructure developments, urban development and conservation activities both by the Government and the private sector. Social protection measures are therefore needed to support and assist evictees in feeding themselves with dignity.

## Additional files

Additional file 1: Interview guide administered to key informants from the study area. (PDF 158 kb)

Additional file 2: Interview guide administered to key informants from the Uganda Human Rights Commission. (PDF 157 kb)

Additional file 3: Questionnaire administered to land evictees (rights-holders). (PDF 224 kb)

## Abbreviations

CESCR: UN Committee on Economic Social and Cultural Rights
CSI: Coping strategy index
CSIS: Coping strategy index score
FGD: Focus group discussion
FIAN: Foodfirst information and action network
GC12: CESCR general comment no. 12 on the right to adequate food
GoU: Government of Uganda
HAZ: Height-for-age Z-score
HFIAS: Household food insecurity access scale
ICESCR: International covenant on economic social and cultural rights
IDDS: Individual dietary diversity score
LAZ: Length-for-age Z score
LC: Local council
RtAF: The human right to adequate food
UHRC: Uganda human rights commission
UNHCHR: United Nations high commissioner for human rights
VGRGT: Voluntary guidelines on the responsible governance of tenure of land, fisheries and forests in the context of national food security
VGRtAF: Voluntary guidelines to support the progressive realization of the right to adequate food in the context of national food security
WAZ: Weight-for-age Z-score
WHZ: Weight-for- height Z-score
