# Supplementary material for: Realization of the right to adequate food and the nutritional status of land evictees: a case for mothers/caregivers and their children in rural Central Uganda
Source: BMC Int Health Hum Rights. 2018 May 24;18:21. doi: 10.1186/s12914-018-0162-6 (PMC5968527; doi:10.1186/s12914-018-0162-6)
Supplement: Supplementary file 1 — Interview guide administered to key informants from the study area. (PDF 158 kb) [file 12914_2018_162_MOESM1_ESM.pdf]

Interview guide administered to Key Informants from the study area

A survey about the realization of the right to adequate food and the nutritional status of land evictees: A case for mothers/caregivers and their children in rural Central Uganda

Respondent's position____

Signature______________________________ Date__________________

1 Do you think the right to adequate food should be recognized as a fundamental human right in Uganda? Please clarify.

2 Objective XXII (a) of the Uganda Constitution talks about the State taking appropriate steps to encourage people to grow and store adequate food. How far do you think this has been achieved?

3 GC 12 of ICESCR obliges States to respect, protect and fulfill the people's right to adequate food. Do you think the government of Uganda is committed to these obligations among land evictees? Please clarify?

4 Are you aware of your tasks and role as a duty-bearer in promoting the human right to adequate food among land evictees? Please clarify

5 Article 237(1) of the 1995 Constitution vests land in the citizens of Uganda, while article 26 of the Constitution guarantees protection from deprivation of property by providing every person a right to own property either individually or in association with others. Do you think land evictees are protected by the constitution? Please clarify?

6 Do you think evictors are following the required procedures of: judicial review before eviction, adequate notice before eviction, compensation for unlawful evictions and criminalization of unlawful evictions? Please clarify?

7 Can you please clarify about the available administrative, quasi-judicial and judicial mechanisms to provide adequate remedies regarding violations of the RtAF among land evictees

8 Do you think land evictees are aware of the available administrative, quasi-judicial and judicial mechanisms?

9 Are these administrative, quasi-judicial and judicial mechanisms accessed and used by land evictees. Please clarify.

9 Is there any legal assistance/aid extended to land evictees to ably take action in order to realise their human right to adequate food in case they feel this right is being violated?

10 If yes, can you please tell us which form of legal assistance is extended to these evictees?

11 Has your institution ever filed any complaints regarding violation of the human right to adequate food by land evictees?

12 If yes, can you tell us what this complaint was about?

11 Are there land evictees in the study area who have received remedy, assistance or reparation after land evictions

12 Was the form of remedy, assistance or reparation after land evictions adequate?

13 Are there any programmes or policies to support land evictees in case of failure to acquire adequate food? Clarify

14 Objectives XXII and XIV of the Uganda Constitution recognizes food and nutrition for all Ugandans. Do you think land evictions have an implication on the food and nutrition security of those evicted? Please clarify

15 What do you think should be done to reduce/stop land evictions?

16 Is there anything about land evictions that you would like to share with me?

Thank you very much for your time!
