# Supplementary material for: Realization of the right to adequate food and the nutritional status of land evictees: a case for mothers/caregivers and their children in rural Central Uganda
Source: BMC Int Health Hum Rights. 2018 May 24;18:21. doi: 10.1186/s12914-018-0162-6 (PMC5968527; doi:10.1186/s12914-018-0162-6)
Supplement: Supplementary file 2 — Interview guide administered to key informants from the Uganda Human Rights Commission. (PDF 157 kb) [file 12914_2018_162_MOESM2_ESM.pdf]

Interview guide administered to Key Informants from the Uganda Human Rights Commission

A survey about the realization of the right to adequate food and the nutritional status of land evictees: A case for mothers/caregivers and their children in rural Central Uganda

Respondent's position____

Signature______________________________ Date___________________

1 Objective XXII (a) of the Uganda Constitution talks about the State taking appropriate steps to encourage people to grow and store adequate food. How far do you think this has been achieved?

2 GC 12 of ICESCR obliges States to respect, protect and fulfill the people's right to adequate food. Do you think the government of Uganda is committed to these obligations among land evictees? Please clarify?

3 Under the 1995 Uganda Constitution, the UHRC is mandated to protect and promote all human rights in Uganda. How far has this been achieved regarding the rights of land evictees in Uganda?

4 Can you please clarify about the available administrative, quasi-judicial and judicial mechanisms to provide adequate remedies regarding violations of the RtAF among land evictees

5 Do you think land evictees are aware of the available administrative, quasi-judicial and judicial mechanisms?

6 Are these administrative, quasi-judicial and judicial mechanisms accessed and used by land evictees. Please clarify.

7 Is there legal assistance/aid extended to land evictees to ably take action in order to realise their human right to adequate food in case they feel this right is being violated?

8 If yes, can you please tell us which form of legal assistance is extended to these evictees?

9 Has your institution ever filed, investigated and adjudicated in court complaints regarding violation of the human right to adequate food by land evictees?

10 If yes, can you tell us what this complaint was about?

11 Are there land evictees in the study area who have received remedy, assistance or

reparation after land evictions

12 Was the form of remedy, assistance or reparation after land evictions adequate?

13 Are there any programmes or policies to support land evictees in case of failure to acquire adequate food? Clarify

14 Objectives XXII and XIV of the Uganda Constitution recognizes food and nutrition for all Ugandans. Do you think land evictions have an implication on the food and nutrition security of those evicted? Please clarify

15 What do you think should be done to reduce/stop land evictions?

16 Is there anything about land evictions that you would like to share with me?

Thank you very much for your time!
