# Supplementary material for: Realization of the right to adequate food and the nutritional status of land evictees: a case for mothers/caregivers and their children in rural Central Uganda
Source: BMC Int Health Hum Rights. 2018 May 24;18:21. doi: 10.1186/s12914-018-0162-6 (PMC5968527; doi:10.1186/s12914-018-0162-6)
Supplement: Supplementary file 3 — Questionnaire administered to land evictees (rights-holders). (PDF 224 kb) [file 12914_2018_162_MOESM3_ESM.pdf]

Questionnaire administered to land evictees (rights-holders)

Introduction and Consent form

Greetings to you. My name is Aziiza Nahalomo, a student from Makerere Univeristy - Kampala. I am conducting a survey about realization of the right to adequate food and the nutritional status of land evictees: A case for mothers/caregivers and their children in rural Central Uganda. This survey is part of the requirements for me to complete my master studies. The information that you provide will be combined with information from other evicted households to help in understanding how evictions affect your human right to adequate food.

The survey is voluntary and you can chose not to answer any individual questions or all of the questions. You may also stop participation at any time. Whatever information you provide will be kept strictly confidential. As part of the study, I will ask you some questions related to land evictions and the realization of the human right to adequate food and also take some measurements of you and one youngest child of 6 months to 5 years in your care. There will be no risks associated with taking measurements using our instruments. Could you please spare some time for the interview that may last around 55 minutes?

Please let me know if anything I have stated is not clear and I will be happy to explain it further to ensure that you understand. Do you agree to take part in this study?

Yes________________ Signature / Thumb print__________

No_________________ Thank you very much!

Land evictions and the realization of the human right to adequate food in Wakiso and Mpigi Districts - rural Central Uganda

Section 1: Household information

Questionnaire number______ District ________County____________<br>Sub county____________ Parish_____________ Village________<br>Household number.________ Allocated household number.______	Date(date/month/year):<br>_____/_____/_____

1.1	**Screening of respondents**<br><br>Are you above 18 years and are you the parent or caregiver of a child 6 - 59 months of age?	If none of the children in the household falls between the age range, respondent should not be interviewed	Yes_1<br><br>No_2

1.2	Who is the head of this household?	Father____________1<br>Mother_________2<br>Any other (please specify)__ 3	
1.3	How many are you in this household?	Total number_________	
1.4	Are you originally born in this area?	Yes___1 No___2	
1.5	If no, how long have you been staying in this area? (Record number of months)		
**Section 2: Questions for understanding the background about evictions**			
2.1	When were you evicted? (Record month and year of eviction)		
2.2	Where were you evicted from?		
2.3	How were you evicted? (Record the description of the respondent)		
2.4	Who carried out the eviction? Tick all appropriate responses	Government__1<br>Military_____2<br>Local investors__3	Police_______4<br>Foreign investor___ 5<br>Others______6
2.5	Did you receive any prior notice or consultation before the eviction?	Yes____1<br>No____2	
2.6	Was a formal authorization for the eviction presented during the eviction?	Yes____1<br>No___2	
2.7	What type of eviction did you face?	All the land___1<br>Part of the land ___2	
2.8	Were any belongings destroyed?	Yes____1 No___2	
2.9	Can you please mention the belongings that were destroyed during the eviction process?	Identification & official papers__1<br>House_____________________2<br>Domestic animals and poultry__3<br>Immovable objects___________4<br>Food______________________5<br>Others_____________________6	
2.10	After evictions, how did you acquire this land and household?	Bought it_____1<br>Given to me___2<br>Just settled there___3	Rented it____4<br>Allocated to me by the evictor_5<br>Others_______6
**Section 3. Effectiveness of the existing complaint and redress mechanisms**			
3.1	In your opinion, have evictions affected your household access to any of the following? Food, school, land, housing facilities and water?	Yes____1<br>No____2	

3.2	Can you please describe how evictions have affected your household's access to food (Record the respondent's response)	
3.3	In your opinion, how would describe the magnitude of the effect?	Bad______1<br>Very bad______2<br>Extremely bad______3<br>Don't know______4<br>Others______________________5
3.4	Are you aware of any complaint / redress mechanisms available for people who have been evicted?	Yes____1<br>No____2
3.5	If yes, can you please mention the available complaint / redress mechanisms known to you (Record respondent's response)	
3.6	Have you ever accessed and used any of them after the eviction?	Yes____1<br>No____2
3.7	If no, why? Record respondent's response	
3.8	If yes, which authority/organ/individual did you report to?	Local council member____1<br>Local area defense__2<br>Resident District Commissioner__3<br>District Land Officers_____4<br>Uganda Police_______5<br>Others___________________6
3.9	Were you assisted adequately?	Yes___1<br>No___2<br>Do not know___3
3.10	Please describe the type of assistance that you received	Food transfers_____1<br>Cash transfers_____2<br>Given alternative land_____3<br>House construction_____4<br>Non repeation______5<br>Apology______6<br>Evictor taken to court/prison__7<br>Others______________________8
3.11	Was any legal assistance/aid extended to you to ably take action in order to realize your human right to adequate food in case you felt this right was being violated?	
3.12	Are there any programmes in this area to support your household in case of failure to acquire adequate food? Clarify	
3.13	Mention the ways in which land evictions affected your human right to adequate food.	Inadequate access to land for agricultural production_1<br>Destruction of crops/food___2<br>Diversion of money to buy food__3<br>Intake of culturally unacceptable food___4<br>Others_______________________ 5
**Section 4: Individual Dietary Diversity Score (IDDS)**		

Please I would like to know the foods you ate yesterday during the day and night. Please describe for me the foods (including snacks) you ate, whether at home or outside home. Please start with the food eaten in the morning.

4.1	Breakfast	Snack	Lunch	Snack	Dinner	Snack

After respondent recall, record all foods eaten above under respective food groups and for any missing food group, ask the respondent if any food item in that group was eaten.

	**Food group**	**Examples**	**Yes_1 No__0**
4.2	Cereals	Corn/maize, rice, wheat, sorghum, millet or any other grains or foods made from these	
4.3	Vitamin A rich vegetables and tubers	Pumpkin, carrots, orange/yellow/purple sweet potatoes	
4.4	White roots and tubers including banana plantain	White potatoes, white yams, white cassava, or other foods made from roots	
4.5	Dark green leafy vegetables	Dark green/leafy vegetables, including wild ones + locally available vitamin-A rich leaves such as dodo, nakati, jobyo, Gobe, cassava leaves, spinach etc.	
4.6	Other vegetables	Other vegetables (e.g. tomato, onion, eggplant) , including wild vegetables	
4.7	Vitamin A fruits	Ripe mangoes, ripe pawpaws, water melon, jackfruit, banana	
4.8	Other fruits	Other fruits, including wild fruits e.g apples, avocados, cashew nuts etc	
4.9	Organ meats	Liver, kidney, heart or other organ meats or blood-based foods	
4.10	Flesh meat	Beef, pork, lamb, goat, rabbit, wild game, chicken, duck, or other birds	
4.11	Eggs	Chicken, duck, eggs	
4.12	Fish	Fresh or dried fish	
4.13	Legumes, nuts and seeds	Beans, peas, lentils, nuts, seeds or foods made from these	
4.14	Milk and milk products	Milk, cheese, yogurt or other milk products	
4.15	Oils and fats	Oil, fats or butter added to food or used for cooking	

Section 5. Coping Strategy Index

**In the past 7 days, if there have been times when you did not have enough food or money to buy food, how many days has your household had to:**		**Frequency: Number of days out of the past seven: (Use numbers 0-7 to answer number of days)**
5.1	Rely on less preferred and less expensive foods?	

5.2	Borrow food, or rely on help from a friend or relative?	
5.3	Purchase food on credit from the market	
5.4	Gather wild fruits or harvest immature crops	
5.5	Consume seed stock held for next season?	
5.6	Send household members to eat elsewhere?	
5.7	Send household members to beg?	
5.8	Limit portion sizes at meal times	
5.9	Restrict consumption by adults in order for small children to eat?	
5.10	Feed working members at the expense of non-working members?	
5.11	Skip entire days without eating?	
5.12	Reduce number of meals eaten in a day?	
**Medium term strategies (Distress)**		
5.13	Depletion of food and seed stocks?	
5.14	Sale of any domestic assets: jewelry, furniture?	
5.15	Sale of productive assets such as livestock, poultry?	
**Long term strategies (Crisis)**		
5.16	Long-term or permanent migration.	
5.17	Pledging land	
5.18	Sale of land	

**Section 6: Household Food Insecurity Access Scale (HFIAS)**			
	Question. **In the past four weeks,**	Response options	Code
6.1	Did you worry that your household (HH) would not have enough food?	1=Yes<br>2= No (skip to next question)	
6.2	How often did this happen?	1 = Rarely (once or twice in the past four weeks)<br>2 = Sometimes (three to ten times in the past four weeks)<br>3 = Often (more than ten times in the past four weeks)	
6.3	Were you or any HH member not able to eat the kinds of foods you preferred because of a lack of resources?	1=Yes<br>2= No (skip to next question)	
6.4	How often did this happen?	1 = Rarely (once or twice in the past four weeks)<br>2 = Sometimes (three to ten times in the past four weeks)<br>3 = Often (more than ten times in the past four weeks)	
6.5	Did you or any HH member have to eat a limited variety of foods due to a lack of resources?	1=Yes<br>2= No (skip to next question)	
6.6	How often did this happen?	1 = Rarely (once or twice in the past four weeks)<br>2 = Sometimes (three to ten times in the	

		past four weeks)<br>3 = Often (more than ten times in the past four weeks)	
6.7	Did you or any HH member have to eat some foods that you really did not want to eat because of a lack of resources to obtain other sources of food	1=Yes<br>2= No (skip to next question)	
6.8	How often did this happen?	1 = Rarely (once or twice in the past four weeks)<br>2 = Sometimes (three to ten times in the past four weeks)<br>3 = Often (more than ten times in the past four weeks)	
6.9	Did you or any HH member have to eat a smaller meal than you felt you needed because there was not enough food?	1=Yes<br>2= No (skip to next question)	
6.10	How often did this happen?	1 = Rarely (once or twice in the past four weeks)<br>2 = Sometimes (three to ten times in the past four weeks)<br>3 = Often (more than ten times in the past four weeks)	
6.11	Did you or any other HH member have to eat fewer meals in a day because there was not enough food?	1=Yes<br>2= No (skip to next question)	
6.12	How often did this happen?	1 = Rarely (once or twice in the past four weeks)<br>2 = Sometimes (three to ten times in the past four weeks)<br>3 = Often (more than ten times in the past four weeks)	
6.13	Was there ever no food to eat of any kind in your HH due lack of resources to get food?	1=Yes<br>2= No (skip to next question)	
6.14	How often did this happen?	1 = Rarely (once or twice in the past four weeks)<br>2 = Sometimes (three to ten times in the past four weeks)<br>3 = Often (more than ten times in the past four weeks)	
6.15	Did you or any HH member go to sleep at night hungry because there was not enough food?	1=Yes<br>2= No (skip to next question)	
6.16	How often did this happen?	1 = Rarely (once or twice in the past four	

		weeks)<br>2 = Sometimes (three to ten times in the past four weeks)<br>3 = Often (more than ten times in the past four weeks)	
6.17	Did you or any HH member go a whole day and night without eating anything because there was not enough food?	1=Yes<br>2= No (skip to next question)	
6.18	How often did this happen?	1 = Rarely (once or twice in the past four weeks)<br>2 = Sometimes (three to ten times in the past four weeks)<br>3 = Often (more than ten times in the past four weeks)	

**Section 7: Anthropometric data**			
**Children (6-59 months) of age**			
7.1	Childs birth date (dd/mm/yr)	____/_______/____	Age in months______
7.2	Sex	M___1<br>F____2	
7.3	Weight(kg)	First measure _______kg	Second measure ____kg
7.4	Presence of bilateral oedema	Yes____1<br>No_____2	
7.5	Height or Length (cm)	First measure ______cm	Second measure __cm
7.6	MUAC (cm)	First measure ______cm	Second measure __cm
**Adults (18 years and above of age)**			
7.7	Sex	M___1<br>F___2	
7.8	Weight (Kg)	First measure ________kg	Second measure _______kg
7.9	Height (cm)	First measure ________cm	Second measure _______cm
**Thank you so much for your positive response, may God bless you and your family.**			
